# *Actinomucor elegans*-fermented *Castanopsis sclerophylla* residue as a functional ingredient for low-glycemic wheat noodles: fermentation-induced functionalization and noodle quality

**DOI:** 10.1016/j.fochx.2026.104724

**Published:** 2026-10-28

**Authors:** Zhenduo Gao, Guanyu Fang, Yaoyu Xu, Yixue Zhou, Fenghua Wu, Zhiping He, Deyang Lin, Baiyi Lu, Xingquan Liu, Yongzhu Zhang

**Affiliations:** a National Key Laboratory for Development and Utilization of Forest Food Resources, College of Food and Health, Zhejiang A&F University, Hangzhou 311300, China; b College of Biosystems Engineering and Food Science, Key Laboratory for Quality Evaluation and Health Benefit of Agro-Products of Ministry of Agriculture and Rural Affairs, Key Laboratory for Quality and Safety Risk Assessment of Agro-Products Storage and Preservation of Ministry of Agriculture and Rural Affairs, Zhejiang University, Hangzhou, China; c School of Food Science and Engineering, Hainan University, Haikou, 570228, China

**Keywords:** *Castanopsis sclerophylla* residue, *Actinomucor elegans*, Wheat noodles, Glycemic index

## Abstract

*Castanopsis sclerophylla* residue (CSR), a starch-processing by-product, is rich in dietary fiber and phenolics but has undesirable sensory attributes and limited food use. This study aimed to functionalize CSR by *A. elegans* fermentation and to evaluate the fermented CSR (FCSR) as a functional ingredient for wheat noodles. Fermentation degraded long-chain macromolecules into small-molecule nutrients, increased soluble dietary fiber (SDF) 2.62-fold and phenolic content by 79.44% relative to native CSR, and markedly altered the volatile profile with increased alcohols and esters, accompanied by improved solubility, water/oil-holding, cation-exchange capacities and antioxidant activity. Putative metabolic pathways were proposed based on the metabolite profiles. At 4% addition, FCSR improved noodle viscoelasticity and cooking quality while maintaining sensory acceptability; resistant starch (RS) increased by 42.18% and predicted glycemic index (pGI) decreased by 16.57% compared with control noodles. These findings suggest that *A. elegans*-fermented CSR is a promising functional ingredient for developing noodles with reduced pGI.

## Highlights

• *elegans* fermentation upgraded CSR into a functional food ingredient.
• Fermentation raised SDF 2.62-fold and phenolic content by 79.44%.
• Fermentation altered volatile profiles and improved physicochemical properties.
• 4% fermented CSR balanced noodle sensory quality and cooking performance.
• 4% fermented CSR raised resistant starch 42.18% and cut predicted GI 16.57%.

## 1 Introduction

Wheat noodles are a popular staple food globally especially in Asia. Starch is the main constituent of native wheat noodles, predominantly in the form of rapidly digestible starch (RDS). The ingestion of this fraction has been associated with a sharp rise in postprandial blood glucose, which may in turn elevates the risks of insulin resistance and obesity (Xiong et al., 2024). Several drugs, such as acarbose and miglitol, are used clinically to inhibit starch hydrolysis. However, their therapeutic use is often constrained by unpleasant side effects, most notably gastrointestinal disturbances including bloating and flatulence (Choi et al., 2010). The strategic incorporation of dietary fiber or fiber-rich ingredients into starch-based formulations is increasingly being studied for its potential to slow digestion and moderate postprandial blood glucose responses (Tian et al., 2025).

*Castanopsis sclerophylla* is a nut-bearing tree widely distributed in the mountainous regions of southern China. Its nut kernel is rich in starch and commonly consumed after steaming or baking, or processed into starch-based foods (Ding et al., 2020). Among plant-processing by-products, CSR is generated in large quantities during *C. sclerophylla* starch processing and is currently used mainly as animal feed or fuel, leading to resource wastage and environmental pollution. CSR was selected in this study because it concentrates most of bioactive components from the nut kernel, making it a potentially valuable substrate for functional ingredient development. It contained about 51% insoluble dietary fiber (IDF), 3.4% SDF, 38% starch, 1.3% phenolics, and 2.2% flavonoids of dry weight (DW). However, CSR's coarse texture and undesirable flavor limit direct use. Microbial fermentation is widely regarded as a cost-effective strategy for modifying the nutritional, flavor and functional properties of agricultural by-products (Wang, Liang, et al., 2025; Zhang et al., 2024). *A. elegans*, a filamentous fungus commonly used in sufu and tempeh fermentation, was chosen because it secretes a broad array of hydrolytic enzymes that can degrade lignocellulosic and proteinaceous matrices, thereby improving fiber solubility, releasing bound phenolics, and modifying volatile profiles (Yin et al., 2020). Despite these promising attributes, the application of *A. elegans* fermentation to functionalize CSR, and the consequent effects of FCSR on the cooking behavior and glycemic response of wheat noodles, have yet to be systematically explored. Fermentation-derived SDF and phenolics are expected to interact with starch and gluten, potentially slowing starch digestion and reinforcing the dough network (Jin et al., 2025; Zhang, Zhou, et al., 2026), which provides a rationale for using FCSR as a functional ingredient in wheat noodles.

The overall objective was to develop FCSR as a functional ingredient for low-GI wheat noodles. Specifically, we (1) characterized the fermentation-induced changes in CSR (nutrients, phenolics, volatiles, metabolites, physicochemical properties) as the basis for its functionality, and (2) evaluated how FCSR addition affects noodle sensory quality, cooking performance, structure, *in vitro* starch digestibility, and pGI. We hypothesized that *A. elegans* fermentation would enzymatically depolymerize the CSR matrix, converting IDF into SDF and liberating bound phenolics. This biotransformation was expected to improve the nutritional, flavor, and physicochemical properties of CSR and enable FCSR to reinforce the starch-gluten network of wheat noodles, thereby retarding starch hydrolysis and lowering the pGI without compromising sensory acceptability.

## 2 Materials and methods

### 2.1 Materials

The dried CSR was provided from Sishiguipu Agricultural Development (Suichang) Co., Ltd. (China). The starch content assay kit (BC0700) was purchased from Beijing Solarbio Science & Technology Co., Ltd. (China). α-Amylase, protease, and amyloglucosidase were purchased from Shanghai Yuanye Bio-Technology Co., Ltd. (China). The strain *A. elegans* (MT229136) was kindly provided by Professor Xiudong Xia from Jiangsu Academy of Agricultural Sciences (Yin et al., 2020). All other reagents were of analytical grade.

### 2.2 CSR fermentation

The strain was reactivated on potato dextrose agar (PDA) at 30 °C for 72 h. Spores were subsequently harvested by scraping and suspended in water. The suspension was filtered through four layers of sterilized gauze to remove residual mycelial debris, and spore concentration was determined by a hemocytometer at 1 × 10^8^ spores/mL. For fermentation, 50 g of CSR was combined with 100 mL of water to obtain an initial moisture content of 69% (w/w) and autoclaved at 121 °C for 20 min. After cooling, 3 mL of the spore inoculum was added to the sterilized substrate and incubated statically at 30 °C for 10 days, with samples withdrawn at 24-h intervals for subsequent analyses.

### 2.3 Fungal enzyme activities

A fresh sample (1.5 g) was extracted with 10 mL of acetate buffer (100 mM, pH 5.0) in a water bath shaker at 30 °C and 200 rpm for 10 min. Following incubation, the extract was centrifuged at 12,000×*g* for 20 min at 4 °C. The clear supernatant was retained for subsequent enzyme activity assays. The activity of amylase, glucosidase, endoglucanase, exoglucanase, and protease were measured in the supernatant according to the protocol established of Zhang et al. (2022). α-Amylase activity was assayed by combining 4 mL of enzyme solution with 2 mL of acetate buffer (0.1 M, pH 4.5) and 2 mL of 1% (w/v) starch solution, after which the mixture was incubated at 37 °C for 60 min. The liberated glucose was quantified by the 3,5-dinitrosalicylic acid (DNS) procedure. One unit of amylase activity corresponded to the quantity of enzyme catalyzing the formation of 1 μg glucose per minute. β-Glucosidase activity was assessed using 0.5 mL of enzyme solution with 4.5 mL of 50 mM citrate buffer (pH 4.8) supplemented with 5 mM *p*-nitrophenyl-β-D-glucopyranoside, followed by incubation at 50 °C for 10 min. Subsequently, Na₂CO₃ was introduced to halt the reaction, and the absorbance was read at 405 nm. One unit of glucosidase activity was defined as the enzyme quantity that catalyzes the liberation of 1 μg *p*-nitrophenol per minute. Protease activity was evaluated by combining 3 mL of enzyme preparation with 3 mL of 1% (w/v) casein solution at 40 °C for 60 min. The reaction was stopped using 10% trichloroacetic acid. After centrifugation, the absorbance was read at 280 nm. One unit of protease activity corresponded to the quantity of enzyme catalyzing the formation of 1 μg tyrosine per minute. Endoglucanase activity was determined by combining 1 mL of enzyme preparation with an equal volume of 0.05 M acetate buffer (pH 5.0) supplemented with 2% (w/v) carboxymethylcellulose, and the mixture was maintained at 50 °C for 30 min. The amount of glucose generated was measured using the DNS procedure. One unit of endoglucanase activity was defined as the enzyme quantity that liberates 1 μg glucose per minute. Exoglucanase activity was evaluated by incubating 1 mL of enzyme preparation with 2 mL of 0.05 M acetate buffer (pH 5.0) in the presence of 100 mg of Whatman No. 1 filter paper at 50 °C for 60 min. Liberated glucose was quantified using the DNS assay. One unit of exoglucanase activity corresponded to the enzyme quantity that catalyzes the release of 1 μg glucose per minute. All experimental results were repeated at least three times.

### 2.4 Contents of starch, protein, dietary fibers, phenolics and total acidity

Starch was quantified using a commercial assay kit (Solarbio, BC0700), while glucose was measured by the DNS method. Total protein was determined *via* the Kjeldahl procedure, and soluble protein content was assessed using Coomassie Brilliant Blue staining. The levels of SDF, IDF, phenolics, and flavonoids were analyzed following protocols provided by Yin et al. (2023). Briefly, dietary fiber was fractionated by dispersing 5 g of freeze-dried sample in 200 mL of MES–TRIS buffer under stirring for 60 min. The dispersion was then subjected to sequential enzymatic hydrolysis, starting with α-amylase (0.5 mL, 10,000 U/mL) at 95 °C for 0.5 h, followed by protease (0.5 mL, 10,000 U/mL) at 45 °C for 0.5 h, and finally amyloglucosidase (0.5 mL, 3300 U/mL) at 60 °C for 0.5 h, with the pH adjusted to 4.5 prior to the final digestion step. After filtration, the retentate was rinsed twice with distilled water preheated to 70 °C and lyophilized to yield IDF, whereas the filtrate was mixed with 95% ethanol at a 1:4 (v/v) ratio and kept at 60 °C to precipitate SDF. For the determination of total phenolic content (TPC) and total flavonoid content (TFC), Freeze-dried samples were subjected to ultrasonic-assisted extraction with 80% (v/v) ethanol in a water bath held at 50 °C for 60 min, and the extracts were clarified by centrifugation prior to TPC and TFC analysis. For TPC, 0.8 mL of the extract was combined with 9.2 mL of deionized water, 2 mL of Folin–Ciocalteu reagent, and 8 mL of 7.5 g/dL Na₂CO₃ solution. After 2 h of incubation in darkness, absorbance was recorded at 760 nm against a gallic acid calibration. For TFC, 6 mL of diluted extract was reacted with 0.3 mL of 5 g/dL NaNO₂ and 0.6 mL of 10 g/dL AlCl₃ for 5 min, followed by the addition of 2 mL of 1 mol/L NaOH for 10 min reaction. Absorbance was measured at 415 nm with rutin as the standard. Total acidity was measured according to Wu et al. (2018). All experimental results were repeated at least three times.

### 2.5 Metabolite profile

Metabolite profiling was carried out using the procedures established by Zhang, Yu, et al. (2026). Fresh specimens (1 g) were extracted with 4 mL of methanol/chloroform/water (2.5:1:1, v/v/v), with ribitol (80 μg/mL) added as an internal standard. The mixture was ultrasonicated at 400 W for 5 min, and after centrifugation, 2 mL of the supernatant was evaporated to dryness under N₂. The dried extract was sequentially derivatized: first by oximation with 200 μL of pyridine containing methoxyamine hydrochloride (20 mg/mL) at 37 °C for 1 h, and then by silylation with 200 μL of *N*-methyl-N-(trimethylsilyl)trifluoroacetamide (MSTFA) containing 1% trimethylchlorosilane (TMCS) at 70 °C for 0.5 h. Following centrifugation and filtration, 1 μL of sample filtrate was analyzed on a gas chromatography–mass spectrometry (GC–MS) system (GCMS-QP2010 Plus, Shimadzu, Japan) fitted with a DB-Wax column (30 m × 250 μm × 0.25 μm, Agilent 122–7032). The column oven was programmed to begin at 45 °C (held for 1 min), then rise to 150 °C over 26 min and maintain for 4 min, subsequently climb to 210 °C over 20 min with a 4 min hold, and finally ramp to 250 °C over 4 min with a 2 min hold. Metabolites were identified against the National Institute of Standards and Technology (NIST) spectral database, with a minimum match threshold of 80%. Metabolite contents were relatively quantified according to peak area using ribitol as the internal standard and expressed as ribitol equivalents (μg/g).

### 2.6 Volatile flavor compounds

Volatile flavor compounds were analyzed using headspace solid-phase microextraction coupled with gas chromatography–mass spectrometry (HS-SPME-GC–MS) (GCMS-QP2010 Plus, Shimadzu, Japan). Briefly, 2 g of fresh sample was incubated at 60 °C for 1 h. A 50/30 μm divinylbenzene/carboxen/polydimethylsiloxane (DVB/CAR/PDMS) SPME fiber was used for headspace extraction. After extraction, the SPME fiber was immediately inserted into the GC injector for thermal desorption. Helium was used as the carrier gas at 1.2 mL/min. The inlet temperature, MS transfer line temperature, and ion source temperature were set at 250 °C, 280 °C, and 230 °C, respectively. The GC–MS conditions were same as described in Section 2.5. Flavor substance contents were relatively quantified according to peak area using 2-octanol as the internal standard and expressed as 2-octanol equivalents (μg/g).

### 2.7 Structural and physicochemical properties

Fourier transform infrared spectroscopy (FT-IR) spectra were recorded with a Nicolet iS20 spectrometer (Thermo Fisher Scientific, USA). Samples were prepared using the conventional KBr pellet method, and spectra were collected in transmittance mode over the wavenumber range of 400–4000 cm^−1^. The crystalline structure was analyzed using X-ray diffraction (XRD) (Bruker D2 PHASER, Germany) at a voltage of 30 kV, current of 10 mA, scanning angle from 5° to 50° and scanning speed of 4°/min. Physicochemical properties, including water-holding capacity (WHC), oil-holding capacity (OHC), water solubility index (WSI), and cation exchange capacity (CEC) were measured following the procedure reported by Wei et al. (2022). WHC was measured by suspending 2 g of sample in 40 mL of distilled water and allowing the mixture to equilibrate for 12 h. Following centrifugation at 4000 rpm for 30 min, the residue was weighed. WHC was calculated as the amount of water retained per 100 g of dry sample (%). OHC was determined by combining 1 g of sample with 8 g of soybean oil, followed by incubation at 37 °C for 4 h. The mixture was centrifuged at 4000 rpm for 15 min, and the residue was weighed. OHC was expressed as grams of oil retained per 100 g of dry sample (%). CEC was assessed by mixing 1 g of sample with 60 mL of 0.1 M HCl at room temperature for 12 h. The sample was then filtered and rinsed until neutral, after which it was treated with 5% NaCl solution. The suspension was stirred and titrated against 0.1 M NaOH with 0.5% phenolphthalein–ethanol solution as indicator. CEC was reported as mmol/g. WSI was assessed by suspending 2 g of sample in 20 mL of distilled water and heating at 90 °C for 30 min. After centrifugation at 2000×*g* for 30 min, the supernatant was dried to constant weight. WSI was expressed as the ratio of dried soluble solids to the initial sample mass (%). All experimental results were repeated at least three times.

### 2.8 Antioxidant capacity

Samples (1 g) underwent double extraction in 40 mL of 80% aqueous ethanol assisted by ultrasonication (50 °C, 30 min). The combined clear liquors obtained after centrifugation were then used to measure antioxidant capacities. The antioxidant capacities, including 2,2-diphenyl-1-picrylhydrazyl (DPPH) radical scavenging activity, 2,2′-azino-bis(3-ethylbenzothiazoline-6-sulfonic acid) (ABTS^+^) scavenging activity, reducing power (RP), and ferric reducing antioxidant power (FRAP), were evaluated according to the study of Yin et al. (2023). The DPPH radical scavenging activity was evaluated by combining 2 mL of sample extract with 2 mL of 0.2 mM DPPH solution in darkness for 30 min. Followed by measurement of the absorbance at 517 nm. The ABTS^+^ radical scavenging capacity was assessed using a working solution prepared by reacting 7 mM ABTS with 2.45 mM potassium persulfate and diluting to an absorbance of approximately 0.70 at 734 nm. Sample extract (2 mL) was then combined with 8 mL of the ABTS^+^ working solution for 6 min before absorbance was read at 734 nm. RP was assessed by mixing 0.2 mL of the extract with 2 mL of PBS (pH 6.6) and 1 mL of 1% K₃[Fe(CN)₆] at 50 °C for 20 min. After adding 1 mL of trichloroacetic acid and centrifugation, 1 mL of the supernatant was combined with 2 mL of 0.1% FeCl₂ for 10 min. Absorbance was then measured at 700 nm. FRAP was determined by mixing 1 mL of the extract with 5 mL of FRAP reagent at 37 °C for 20 min. Absorbance was then measured at 593 nm. All experimental results were repeated at least three times.

### 2.9 Noodle processing

Freeze-dried CSR and FCSR powders proportionally replaced wheat flour at the addition levels of 1%, 2%, 4%, and 6% by weight (w/w substitution). Then, 200 g of the mixture was mixed with 70 mL of distilled water and kneaded in an automatic dough mixer for 30 min. Noodle sheets were then produced using a combined rolling and cutting machine. The dough was passed through the rollers and extruded repeatedly for 20 cycles to achieve proper consistency. Finally, the dough was flattened to a thickness of 2 mm and sliced into strips of 15 cm in length.

### 2.10 Color measurement

Noodle samples were laid out in a compact monolayer across a flat dish. A colorimeter (NR110, 3nh, China) was then employed to capture readings at three separate sites on the surface, yielding the L, a, and b color-space values. These parameters were subsequently used to derive the whiteness index, designated as A, through the prescribed equation.

$$A=100-\sqrt{\left[\;{\left(100-L^{*}\right)}^{2}+{a^{*}}^{2}+{b^{*}}^{2}\right]}$$

### 2.11 Cooking performance

The uncooked noodles were weighed before cooking and recorded as W. The noodles were boiled in a pot of water until the opaque central core had just disappeared. After cooking, they were lifted out, transferred to a colander, and rinsed with deionized water for 30 s, followed by a 5-min draining period. The drained noodles were then weighed to obtain the value W_1_. Afterward, the same sample was dried until its mass became constant, recorded as W₂. The two quality indicators including water uptake and solid loss during boiling were determined using the following equations:

$$\text{Solid loss}\;\left(\%\right)=\left[\left(W_{0}-W_{2}\right)/W_{0}\right]\times 100$$

where W_0_ denotes the initial dry weight of the uncooked noodles;

$$\text{Water uptake}\;\left(\%\right)=\left[\left(W_{1}-W_{0}\right)/W_{0}\right]\times 100$$

where W_0_ represents the pre-cooking wet weight of the sample.

### 2.12 Rheological property

The rheological properties of noodle dough were measured using a rotational rheometer (HR-1, TA Instruments, USA) based on the study of Huang et al. (2022). After resting for 20 min, the dough samples were placed at the center of the rheometer plate. Measurements were conducted at 25 °C using a parallel plate geometry with a plate diameter of 40 mm and a gap of 1 mm. Frequency sweep tests were performed at a fixed strain of 0.5% over a frequency range of 0.1–100 Hz. The storage modulus (G′), loss modulus (G″), and loss tangent (tanδ) were recorded as functions of frequency.

### 2.13 Sensory assessment

Sensory characterization was carried out by a 30-member expert panel. Seven sensory criteria composed of color, firmness, elasticity, adhesiveness, aroma, taste, and overall acceptability were each scored on a scale from 1 to 10. The freshly made noodles were cooked in boiling water for 3 min, drained, and offered warm to the assessors. Evaluations occurred immediately upon production. The sensory evaluation protocol was reviewed and approved by the Life Science and Medical Ethics Committee of Zhejiang A&F University (record No. SMYX-2026-013). All panelists voluntarily participated in the sensory evaluation and provided informed consent prior to participation.

### 2.14 Scanning electron microscopy (SEM) observation

Fresh noodles were cooked, immediately cooled with water and blotted with absorbent paper to remove excess surface water. Samples were then fixed in 5% paraformaldehyde solution for 2 h. Then, samples were freeze-dried and fractured in liquid nitrogen. The fractured samples were sputter-coated with gold and observed using a scanning electron microscope (SEM, GeminiSEM 360, ZEISS, Germany) at 3.00 kV and 200-fold magnification.

### 2.15 *In vitro* digestion and pGI value

The *in vitro* digestion and pGI value were analyzed followed the procedure described by Zhang, Yu, et al. (2026) and Zhang, Zhou, et al. (2026). In brief, 400 mg of powdered samples were introduced into 10 mL of sodium acetate buffer (pH 5.2) supplemented with α-amylase (100 U/mL) and α-glucosidase (24 U/mL). The suspension was incubated at 37 °C in a shaking water bath. At 20-min intervals, 0.4-mL portions were withdrawn and immediately mixed with 4 mL of 95% ethanol. After centrifugation, the supernatants were assayed for glucose content using the DNS reagent. The hydrolysis index (HI) was calculated as the percentage ratio of the area under the starch hydrolysis curve (AUC, 0–180 min) of the sample to that of standard white bread used as the reference food, following the HI definition of Granfeldt et al. (1992): HI (%) = (AUC _sample_/AUC_white bread_) × 100. The pGI value was calculated using the equation: pGI = 8.198 + 0.862HI.

### 2.16 Statistical analysis

All experimental data were expressed as mean values derived from a minimum of three independent replicates. To evaluate intergroup differences, a one-way analysis of variance (ANOVA) was performed, followed by Duncan's multiple-range test for post-hoc comparisons, using SPSS 16.0. Statistical significance was set at a threshold of *p* < 0.05. Pearson correlation analysis was conducted to investigate the correlations among enzyme activities, nutritional components and physicochemical properties as well as the correlations among enzyme activities, phenolics and antioxidant activity. The data of metabolite composition was analyzed by Principal component analysis (PCA) using Origin and PERMANOVA analysis. PERMANOVA was performed in R (vegan and permute packages) using Euclidean distances after mean-centering and unit-variance scaling, with all 719 non-identity permutations. Orthogonal partial least squares discriminant analysis (OPLS-DA) was performed on the flavor compound composition data using Simca 14.1. Model validation was carried out through permutation testing with 200 iterations. The goodness of fit was assessed primarily by Q2 (predictive capability of the model) and R2 (cumulative interpretive capacity of the model).

## 3 Results and discussion

### 3.1 Trends of enzyme activities during fermentation

As shown in Fig. 1A, *A. elegans* exhibited vigorous growth on CSR during fermentation, producing abundant white hyphae. *A. elegans* secreted an abundant array of enzymes, including α-amylase, protease, cellulase, and β-glucosidase, into substrate to support its growth (Fig. 1B-E). The activities of α-amylase, protease, endoglucanase and exoglucanase exhibited similar trends during fermentation, characterized by an initial increase followed by a gradual decrease. The β-glucosidase activity significantly increased and then remained constant during fermentation. These extracellular hydrolases are likely responsible for degrading macromolecular nutrients such as starch, protein, and cellulose into oligosaccharides, glucose, peptides, amino acids, organic acids, and other substances, which not only improved the nutritional composition of the CSR but also affected its structural and physicochemical properties (Chen et al., 2020).

**Fig. 1:**
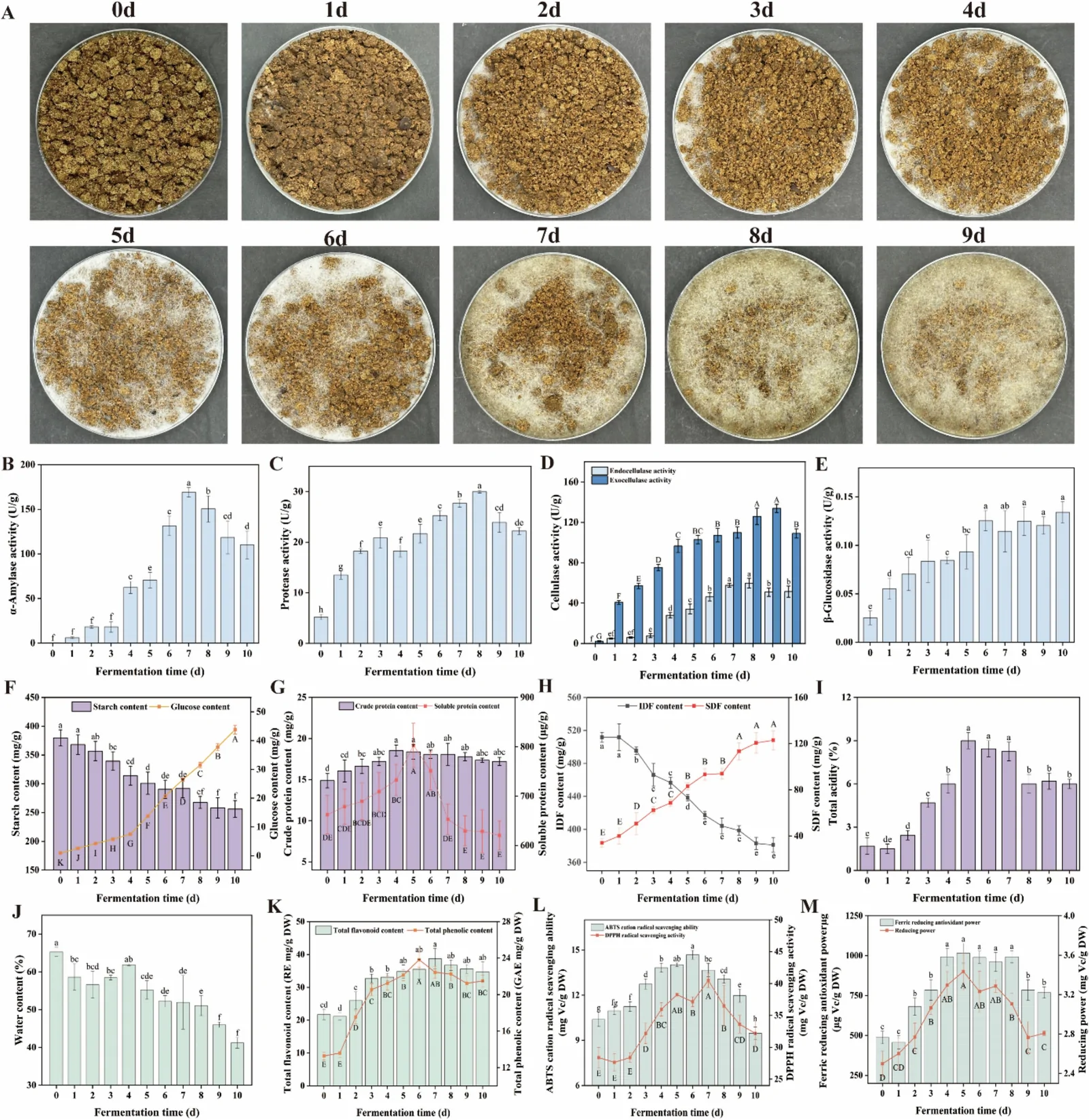
Changes on enzyme activity, main nutrients, and antioxidant activity of CSR during fermentation. (A) Morphologic observation; (B) amylase; (C) protease; (D) cellulase. (E) Glucosidase. (F) Starch and glucose contents. (G) Crude and soluble proteins. (H) Dietary fibers. (I) Total acidity; (J) water content; (K) phenolic and flavonoid contents; (L) ABTS•^+^ and DPPH radical scavenging ability; (M) RP and FRAP.

### 3.2 Changes in the nutritional components of CSR during fermentation

As demonstrated in Fig. 1F, the CSR starch content significantly decreased by 32.54% after 10 d fermentation, leading to an obvious increase in glucose content in FCSR. The crude protein content increased from 14.88 mg/g to 18.52 mg/g at 4 d and then remain constant (Fig. 1G). This increase is most likely attributable to the accumulation of microbial protein and the loss of carbohydrates during fermentation (Canoy et al., 2024). The soluble protein content showed a trend of first increasing and then decreasing. The bound proteins with high molecular weight are presumed to be hydrolyzed by proteases derived from *A. elegans* into soluble proteins. Subsequently, they may further be utilized by microorganisms or degraded into free amino acids, small peptides, and other small-molecule nitrogen-containing compounds (Ren et al., 2025). IDF was the dominant fraction in CSR, accounting for 51.17% DW at 0 d, but decreased by 25.5% at 10 d (Fig. 1H). Conversely, SDF content exhibited a 2.62-fold increase after fermentation for 10 d relative to native CSR, suggesting a substantial conversion of IDF into soluble forms during fermentation. As reported by Cui et al. (2024) in wheat bran fermentation, enzymes like cellulase and xylanase can break down the rigid plant cell wall structure, which may facilitate IDF degradation and solubilization and contribute to the observed increase in SDF. This increase in SDF is expected to contribute to water binding and starch-gluten interactions in noodle dough, which is examined in the following Sections. Unlike the particulate IDF, SDF exhibits superior regulatory effects on blood glucose and lipids (Gill et al., 2021). The total acid content of CSR increased initially during fermentation and then decreased (Fig. 1I). Additionally, the water content suffered a decreased trend during fermentation (Fig. 1J).

### 3.3 Alterations in phenolic content and antioxidant activity during fermentation

The phenolic content of FCSR rose steadily over the initial 6 days of fermentation, reaching a maximum of 23.83 gallic acid equivalents (GAE) mg/g DW, a 79.44% increase relative to CSR, before exhibiting a modest decline thereafter (Fig. 1K). Flavonoid levels followed a comparable trajectory. These observations suggested that fermentation promoted the release of the bound phenolics from CSR matrix. In plant tissues, phenolics are frequently sequestered within cell wall components or conjugated to sugar moieties. Nevertheless, the hydrolytic enzymes generated during fermentation, such as α-amylase, β-glucosidase, and cellulase, are capable of cleaving glycosidic and ester bonds embedded in the wall architecture, which may contribute to the mobilization of bound phenolics (Wang, Li, et al., 2025). The increased phenolic content may also contribute to starch digestion modulation in FCSR-fortified noodles in the following sections. Antioxidant activity is the most outstanding bioactivity of phenolics and flavonoids. Hence, four antioxidant assays were performed to evaluate the changes of CSR’ antioxidant activity. The DPPH radical scavenging activity, ABTS^+^ radical scavenging activity, RP, and FRAP all significantly increased during the early stages of fermentation and then decreased (Fig. 1L-M). Interestingly, the peak times of the four antioxidant indicators did not completely coincide. This variation might be related to changes in the species of phenolic compounds, hydrogen-donating ability, and aromatic ring electron delocalization capacity during fermentation (Zhao et al., 2024). The decline in phenolic content and antioxidant capacity during late-stage fermentation (from Day 5–6 onward) likely reflected a shift in the balance between phenolic release and oxidative consumption. As readily available carbon sources become depleted, fungal metabolism shifts, and oxidative enzymes such as polyphenol oxidases and peroxidases can catalyze the oxidation of free phenolics into quinones, which subsequently undergo polymerization or condensation with proteins, rendering them undetectable by conventional colorimetric assays (Xiao et al., 2022). In addition, microbial degradation of phenolic compounds *via* ring cleavage or further metabolic transformation may exceed the rate of enzymatic release from bound forms once the easily hydrolyzable fractions of the substrate have been exhausted (Zhao et al., 2024). The high Pearson correlation are consistent with the possibility that fungal enzyme-induced phenolic release contributed to the enhanced antioxidant activity of CSR (Fig. S1).

### 3.4 Flavor component analysis

The FCSR at 5 d was used to analyze flavor components according to the sensory evaluation results (Supplementary Table 1). 77 volatile substances were totally identified in both CSR and FCSR (Table 1). The established OPLS-DA model was validated by its high parameters (Q^2^ = 0.993, R^2^X = 0.734, R^2^Y = 1.0), and the score plot exhibited a clear separation between the two groups (Fig. 2A). Model reliability was confirmed by the negative Q^2^ regression intercept along with the elevated original Q^2^ value (Fig. 2B). Furthermore, S-plot and variable importance in projection (VIP) analysis (threshold >1) singled out 44 differential flavor markers (Fig. 2C-D), indicating that fermentation resulted in substantial changes in the volatile composition.

**Table 1:** Flavor compounds detected *via* HS-SPME-GC–MS analysis in CSR and FCSR.

Category	No.	Compounds	Relative content (2-Octanol equivalent μg/g)	
			CSR	FCSR
*Hydrocarbons*	1	2,2-Dimethyldecahydronaphthalene	67.75 ± 1.16	N.D.
	2	Hexamethyloctahydro-1H-indene	46.05 ± 1.36^a^	6.51 ± 7.09^b^
	3	Phenanthrene	46.79 ± 1.42	N.D.
	4	Cyclopentane	64.12 ± 39.03	N.D.
	5	Pyrenehexadecahydro	24.35 ± 22.36^a^	11.56 ± 3.04^a^
	6	Heneicosane	15.71 ± 6.23^a^	5.49 ± 2.79^a^
	7	Naphthalene	N.D.	35.16 ± 24.36
	8	2-Methyl-5-pentane	88.90 ± 77.31^a^	22.60 ± 22.66^a^
	9	1,1-Bicyclopentyl	27.07 ± 23.45	N.D.
	10	Dodecane	318.12 ± 50.56^a^	49.36 ± 27.71b
	11	Heptadecane	172.59 ± 30.44^a^	39.01 ± 4.21b
	12	Tridecane	59.62 ± 49.14^a^	34.44 ± 17.96a
	13	Decane	58.93 ± 50.82	N.D.
	14	Hexadecane	629.08 ± 133.06^a^	73.09 ± 50.61^b^
	15	Tetratetracontane	116.68 ± 25.41^a^	7.07 ± 4.46^b^
	16	Pentadecane	541.44 ± 17.41^a^	168.65 ± 150.45^b^
	17	Tetradecane	209.96 ± 55.49^a^	32.53 ± 7.28^b^
	18	1-Chlorooctadecane	45.07 ± 16.37	N.D.
	19	Heptacosane	46.39 ± 23.30	N.D.
	20	Eicosane	7.96 ± 6.10^a^	4.79 ± 3.56^a^
	21	Cyclohexane	88.19 ± 22.41	N.D.
	22	4-Methyldocosane	15.59 ± 18.38	N.D.
	23	2-Bromotetradecane	29.31 ± 34.32^a^	14.45 ± 8.45^a^
	24	5-Methyl-5-propylnonane	30.60 ± 45.29^a^	10.27 ± 12.55^a^
	25	4-Cyclohexyltridecane	13.06 ± 11.62	N.D.
	26	Tridecane, 2-methyl-	N.D.	13.32 ± 3.11
	27	1-Chloroeicosane	N.D.	19.26 ± 0.70
	28	3-Dodecyne	6.57 ± 1.63	N.D.
		Total hydrocarbons	2769.89 ± 131.08^a^	547.58 ± 218.53^b^
*Aldehydes*	29	2-Octenal	20.39 ± 1.05	N.D.
	30	Nonanal	141.44 ± 30.65^a^	17.77 ± 6.70^b^
	31	Decanal	63.19 ± 12.61^a^	8.11 ± 4.01^b^
	32	Benzaldehyde	21.90 ± 6.30	N.D.
	33	2-Decenal	79.33 ± 4.61	N.D.
	34	Undecanal	19.76 ± 0.91	N.D.
	35	5-Hexyl-2-furaldehyde	15.92 ± 1.36	N.D.
	36	Hexadecenal	63.49 ± 27.76	N.D.
		Total aldehydes	425.42 ± 51.24^a^	25.88 ± 8.66^b^
Alcohols	37	1-Octanol	7.93 ± 0.18a	10.33 ± 3.06a
	38	Oleyl alcohol	30.15 ± 20.42^a^	14.63 ± 12.18^a^
	39	1-Heptanol	10.15 ± 1.23	N.D.
	40	1-Nonanol	6.60 ± 2.75	N.D.
	41	2-Isopropyl-5-methyl-1-heptanol	4.81 ± 0.05	N.D.
	42	1-Heptacosanol	41.00 ± 27.78	N.D.
	43	Guaiol	N.D.	23.07 ± 5.97
	44	2-Adamantanol	N.D.	8.89 ± 2.03
	45	4-Hexadecanol	N.D.	9.66 ± 6.76
	46	5-Hexenol, 5-methyl-	N.D.	33.17 ± 11.91
	47	3-Pentenol	N.D.	5.39 ± 0.94
	48	Methoxycyclohexanol	N.D.	7.15 ± 1.04
	49	1-Dodecanol	N.D.	13.05 ± 3.18
	50	1-Decanol, 2-hexyl-	N.D.	9.44 ± 3.22
	51	2-Hexyl-1-octanol	N.D.	9.64 ± 6.18
	52	1-Heptanol, 2,4-diethyl-	N.D.	8.28 ± 0.38
	53	Phenylethyl alcohol	N.D.	2.61 ± 0.97
	54	Phytol	N.D.	29.32 ± 9.95
	55	1,5-Pentanediol, 3-methyl-	N.D.	3.21 ± 2.61
		Total alcohols	100.63 ± 52.40^a^	187.86 ± 70.35^a^
*Esters*	56	Linoleic acid ethyl ester	N.D.	3.61 ± 0.12
	57	Cyclopropanetetradecanoic acid, methyl ester	N.D.	17.43 ± 1.19
	58	1,2-Benzenedicarboxylic acid, 2-methylpropyl ester	5.98 ± 1.48	N.D.
	59	9-Octadecen-12-ynoic acid, methyl ester	N.D.	7.20 ± 1.49
	60	Hexadecanoic acid ethyl ester	N.D.	8.38 ± 3.05
	61	Sulfurous acid, 2-propyl tridecyl ester	N.D.	9.14 ± 1.62
	62	9-Octadecenoic acid ethyl ester	N.D.	3.64 ± 0.66
	63	Acetic acid, hexadecyl ester	N.D.	13.11 ± 3.23
	64	Dichloroacetic acid-4-hexadecyl ester	N.D.	13.72 ± 3.24
	65	Tetradecanoic acid, ethyl ester	N.D.	3.82 ± 0.86
		Total esters	5.98 ± 1.48^b^	80.06 ± 6.90^a^
*Ketones*	66	3-Heptanone	4.12 ± 3.57	N.D.
	67	3-Octenone	5.92 ± 0.22	N.D.
	68	2-Dodecanone	8.81 ± 7.67	N.D.
	69	Cyclohexanone	20.71 ± 2.09	N.D.
	70	9-Octadecanone	13.74 ± 0.72	N.D.
	71	Cyclopentanone	14.78 ± 12.82	N.D.
	72	3-Nonenone	45.44 ± 39.36	N.D.
	73	2-Piperidinone	35.97 ± 11.92^a^	13.70 ± 11.16^a^
	74	2-Pentadecanone	12.42 ± 2.82^a^	4.20 ± 2.11^b^
	75	2-Octanone	N.D.	30.48 ± 15.50
		Total ketones	161.92 ± 33.03^a^	48.38 ± 12.56b
*Furans*	76	2-Pentyl-furan	41.61 ± 5.22	N.D.
*Phenols*	77	(1,1-dimethylethyl) Phenol	111.13 ± 5.30^a^	3.20 ± 2.91^b^

Means with different small letters are significantly different (*p* < 0.05). N.D., not detected. Relative content is expressed as 2-octanol equivalents.

**Fig. 2:**
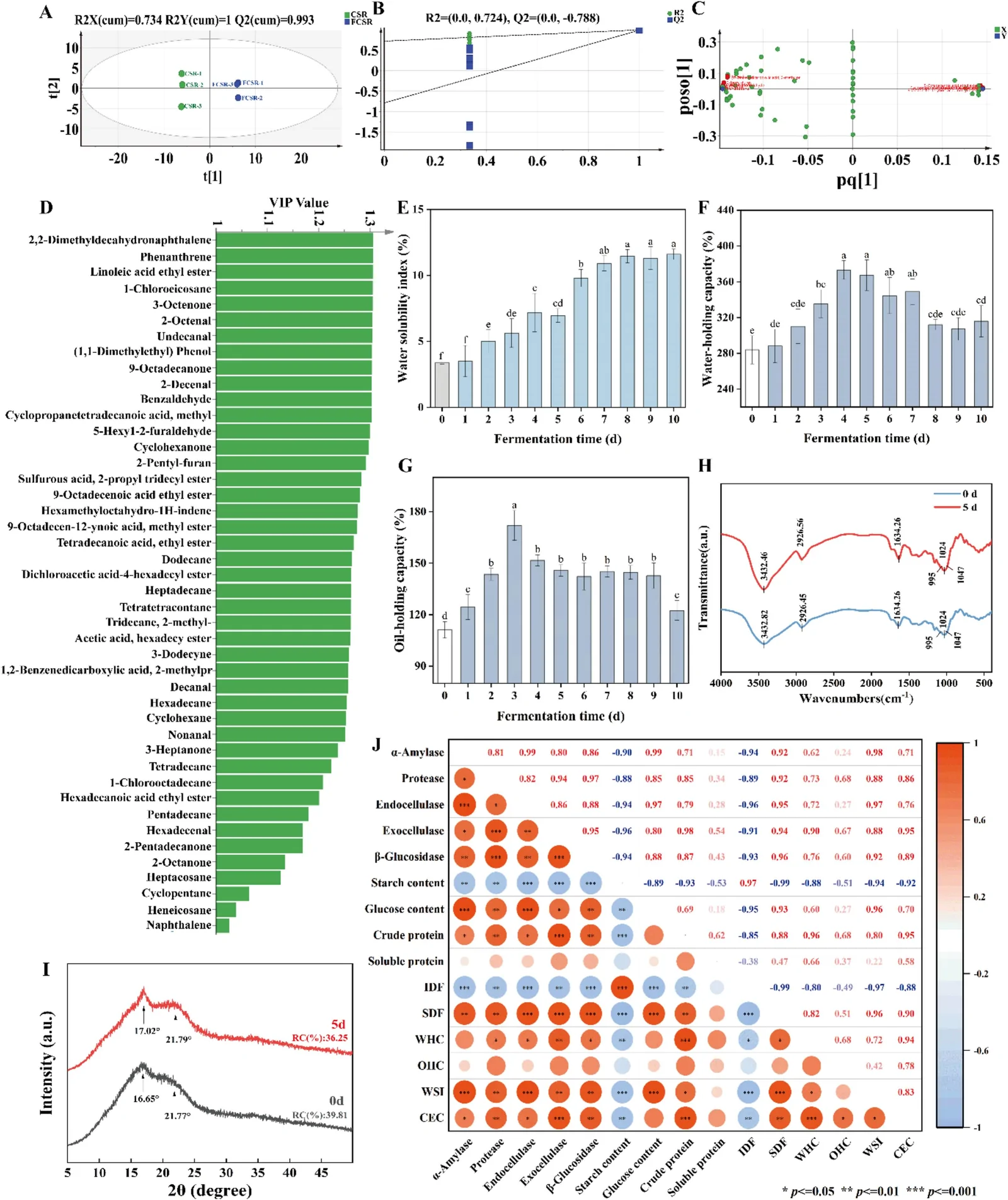
Effects of fermentation on the composition of flavor compounds and physicochemical properties of CSR. (A) OPLS-DA score plot; (B) Permutation plot; (C) S-plot; (D) VIP plot (>1); (E) Water solubility index; (F) Water-holding capacity; (G) Oil-holding capacity; (H) FT-IR analysis; (I) XRD analysis; (J) The correlation analysis among enzyme activities, nutrient contents and physicochemical properties.

The volatile compounds were categorized into seven classes (Table 1). In CSR, hydrocarbons (2769.89 μg/g) predominated, followed by aldehydes (425.42 μg/g) and ketones (161.92 μg/g). After fermentation, the profile shifted markedly, with hydrocarbons (547.58 μg/g), alcohols (187.86 μg/g), and esters (80.06 μg/g) emerging as the three most abundant groups in FCSR. The hydrocarbon content comprising both saturated and unsaturated alkanes fell by 80.23% after fermentation. Despite being the most prevalent group, alkanes are generally thought to play a minor role in flavor formation (Xia & Li, 2018). A significant decrease of 93.92% in the total aldehyde level was observed after fermentation. Aldehydes have sometimes been associated with lower food acceptability and may contribute to off-notes (Melgarejo et al., 2011); however, their actual sensory impact in CSR/FCSR was not assessed in this study. The conversion of aldehydes to alcohols *via* catalytic reactions has been reported to occur during microbial metabolism (Di Cagno et al., 2009). Consistent with this possibility, a significant elevation in alcohol concentrations was observed following fermentation. Alcohols have been reported to contribute to floral and fruity notes (Fawole & Opara, 2014), although their specific sensory role in FCSR remains to be verified. In contrast to CSR, where only a single ester was identified, the total ester content in FCSR increased by 12.39-fold after fermentation. Esters, which are generally formed from alcohols and carboxylic acids, are characterized by low odor thresholds and have been associated with sweet, floral, or fruity notes (Wang, Liang, et al., 2025). This substantial increase may be favorable for the aroma profile of CSR, given the low odor thresholds and generally pleasant notes of esters. Fermentation also significantly decreased the level of ketones, which are generally derived from amino acid degradation or the oxidation of unsaturated fatty acids (Yin et al., 2023). 2-Pentylfuran was detected in CSR and has previously been reported to exert adverse effects at certain exposure levels (Liu, Gu, et al., 2022); its actual health relevance in CSR requires further assessment. Taken together, these results indicate that *A. elegans* fermentation markedly changed the volatile composition of CSR in a direction generally associated with more desirable aroma attributes.

### 3.5 Metabolite composition analysis

A total of 49 metabolites were detected *via* GC–MS analysis across CSR and FCSR at 5 d, and these compounds fell into nine categories: carbohydrates, alcohols, fatty acids, amino acids, organic acids, inorganic acid, esters, phenols, and others (Table 2). PCA of the metabolite profiles showed that PC1 and PC2 accounted for 77.9% and 11.7% of the total variance, respectively (Fig. S2). CSR samples formed a tight cluster, whereas FCSR samples showed greater within-group dispersion. The larger within-group dispersion of FCSR is not unexpected for solid-state fermentation samples, in which localized differences in mycelial growth, moisture, oxygen availability, and substrate accessibility can generate batch-to-batch metabolic heterogeneity. PERMANOVA indicated that the between-group difference accounted for a large proportion of the total variance (R^2^ = 0.776), although the difference was not statistically significant at the 0.05 level (pseudo-F = 13.893, *p* = 0.100), which is likely attributable to the limited sample size (*n* = 3 per group). Hierarchical cluster analysis (Fig. S3) and the pronounced changes in individual metabolites (Table 2) further supported a fermentation-driven shift in metabolite composition.

**Table 2:** Metabolites detected *via* GC–MS analysis in CSR and FCSR.

Metabolites	Relative content (Ribitol equivalent μg/g)		Metabolites	Relative content (Ribitol equivalent μg/g)	
	CSR	FCSR		CSR	FCSR
**Carbohydrates**			Total	95.89 ± 15.84^b^	363.74 ± 70.85^a^
Glucose	N.D.	26.07 ± 0.47	**Amino acids**		
Lactose	N.D.	4.13 ± 0.22	Proline	N.D.	7.29 ± 7.97
Mannobiose	22.94 ± 2.05	N.D.	Glycine	3.16 ± 0.75^b^	57.95 ± 44.40^a^
Arabinose	55.24 ± 12.75	N.D.	Aspartic acid	N.D.	7.63 ± 1.96
Fructose	3.74 ± 1.19^a^	6.98 ± 1.92^a^	Total	3.16 ± 0.75^b^	72.86 ± 38.94^a^
Total	81.92 ± 12.67^a^	37.18 ± 1.50^b^	**Fatty acids**		
**Alcohols**			Hexadecanoic acid	N.D.	1.43 ± 0.16
Glycerol	N.D.	22.15 ± 2.14	Palmitic acid	116.82 ± 40.24	N.D.
Pentitol	N.D.	4.55 ± 0.25	Total	116.82 ± 40.24^a^	1.43 ± 0.16^b^
1,1-Dimethylethanol	5.63 ± 0.97^a^	8.98 ± 4.67^a^	**Esters**		
Arabitol	3.31 ± 0.39^b^	19.90 ± 4.42^a^	Formic acid, 2-propenyl ester	N.D.	66.97 ± 7.14
Mannitol	N.D.	0.84 ± 0.29	Carbonocyanidic acid, ethyl ester	2.90 ± 0.72^b^	8.91 ± 1.34^a^
1-Propanol	2.36 ± 3.15^b^	18.39 ± 12.22^a^	Hexanedioic acid, 2-ethylhexyl ester	6.43 ± 6.00^a^	6.58 ± 3.64^a^
2,4-Pentadienol	N.D.	12.94 ± 0.81	2-Methylpentanoic acid ester	N.D.	4.69 ± 1.54
2-Hydrazinoethanol	N.D.	33.86 ± 14.50	Sulfurous acid, 2-ethylhexyl hexyl ester	N.D.	1.06 ± 1.25
3-Furanmethanol	N.D.	8.04 ± 7.60	Total	9.33 ± 6.37^b^	88.21 ± 3.28^a^
1,2-Ethanediol	N.D.	4.28 ± 5.62	**Inorganic acids**		
Ethanol	N.D.	455.79 ± 84.12	Phosphoric acid	N.D.	1.82 ± 0.19
Isobutanol	N.D.	2.89 ± 0.84	**Phenols**		
Isopropyl alcohol	N.D.	215.84 ± 30.42	Phenol, 2-methoxy-	N.D.	2.41 ± 1.36
Inositol	N.D.	2.18 ± 0.18	Eugenol	N.D.	2.32 ± 0.56
Total	11.30 ± 4.42^b^	810.63 ± 99.15^a^	2,4-Di-tert-butylphenol	7.39 ± 6.62^b^	26.05 ± 1.32^a^
**Organic acids**			1H-Indenol, 2,3-dihydro-1-methoxy-	N.D.	6.72 ± 1.30
2-Amino-2-methyl-butenoic acid	7.19 ± 1.66	N.D.	Total	7.39 ± 6.62^b^	37.50 ± 1.49^a^
Acetic acid	36.83 ± 13.31^b^	123.92 ± 88.91^a^	**Others**		
Butyric acid	44.87 ± 5.48^b^	127.56 ± 51.78^a^	2-Hydroxy-butyrolactone	N.D.	50.22 ± 43.89
Carboxylic acid	N.D.	4.12 ± 0.88	1H-Pyrazole-1-carboximidamide	N.D.	2.15 ± 0.17
Hexanoic acid	N.D.	0.82 ± 0.06	Acetaldoxime	13.17 ± 10.18	N.D.
Pentanoic acid	7.00 ± 0.73^a^	15.98 ± 6.72^a^	1-Butenyne	N.D.	511.51 ± 67.74
Gluconic acid	N.D.	4.63 ± 0.91	Tetrahydrofuran	75.73 ± 13.63^a^	4.16 ± 0.44^b^
Propanoic acid	N.D.	83.64 ± 77.13	Urea	24.26 ± 5.84^a^	16.44 ± 4.96^a^
Malic acid	N.D.	3.07 ± 0.15	Total	113.17 ± 28.82^b^	584.48 ± 93.09^a^

Means with different small letters are significantly different (*p* < 0.05). N.D., not detected. Relative content is expressed as ribitol equivalents.

Fermentation exerted a profound impact on the carbohydrate profile, which predominantly consisted of mono- and disaccharides (Table 2). Among these, glucose, lactose, and tagatose showed marked increases following fermentation, likely resulting from the breakdown of long-chain carbohydrates by glycoside hydrolases produced by *A. elegans* (Zhang et al., 2022). In contrast, galactose and mannobiose were completely depleted, suggesting their possible channeling into the tricarboxylic acid (TCA) cycle or conversion to sugar alcohols. This inference is consistent with the concurrent elevation of pentitol, arabitol, mannitol, and inositol. The increased glycerol may result from glycerophospholipids hydrolysis, as indirectly supported by the increased hexadecanoic acid and palmitic acid. Several alcohols, namely 3-furanmethanol, ethanol, isobutanol, and isopropyl alcohol, were newly generated during fermentation. Although alcohols have a high threshold, their esterification with organic acids may contribute to the aroma profile of CSR (Wang, Liang, et al., 2025). Meanwhile, the total amino acid content in FCSR exhibited a 22.06-fold increase compared with CSR, which could be largely ascribed to proteolytic activity or transamination processes. Additionally, the elevated phosphate level in FCSR is likely attributable to phytase-mediated degradation of phytic acid (Yin et al., 2020), although phytase activity was not directly measured here. The organic acid profile of FCSR was far more abundant than that of CSR, in line with its greatly elevated total acidity (Fig. 1I). Notable increases were detected in short-chain fatty acids (SCFAs), specifically acetic, butyric, propanoic, and hexanoic acids, after fermentation. These SCFAs are likely produced from dietary fiber breakdown and have been reported to improve gut microbiota composition, immune modulation, and inflammatory signaling (Gill et al., 2021). Whether such effects occur *in vivo* after FCSR consumption remains to be established. The increased acetic, propanoic, and hexanoic acid levels were consistent with pathways previously described in filamentous fungi, including pyruvate decarboxylation to acetyl-CoA or the Wood–Ljungdahl pathway for acetic acid, the succinate/propanediol/acrylate routes for propanoic acid, and reverse β-oxidation for hexanoic acid (Akhtar et al., 2022). However, these pathways were not experimentally verified in the present study and should be regarded as putative. The increased gluconic acid may originate from glucose oxidation; gluconic acid is widely used as a food acidulant and has been associated with a mild sour taste, although its sensory contribution in FCSR was not evaluated. The elevated malic acid level is consistent with an enhanced TCA cycle during fermentation, although direct flux evidence is lacking. These SCFAs, together with the other newly generated organic acids (malic, gluconic, and pentanoic acids), accounted for the markedly elevated total acidity of FCSR (Fig. 1I). Because acetic, propanoic, and butyric acids have low odor thresholds and typical sour notes, in subsequent experiments, adding FCSR into noodles might influence the product's taste score through alterations in acidity. The total level of esters in FCSR significantly increased by 8.45 times compared with control. Esters are generally characterized by low odor thresholds and have been associated with fruity and floral notes (Li et al., 2022); their actual contribution to FCSR aroma requires sensory validation. The increased phenol content was due to the liberation of bound phenolics as describe above. To provide a comprehensive overview of the biochemical events underlying CSR fermentation, the putatively activated metabolic pathways were compiled and are presented in Fig. 3.

**Fig. 3:**
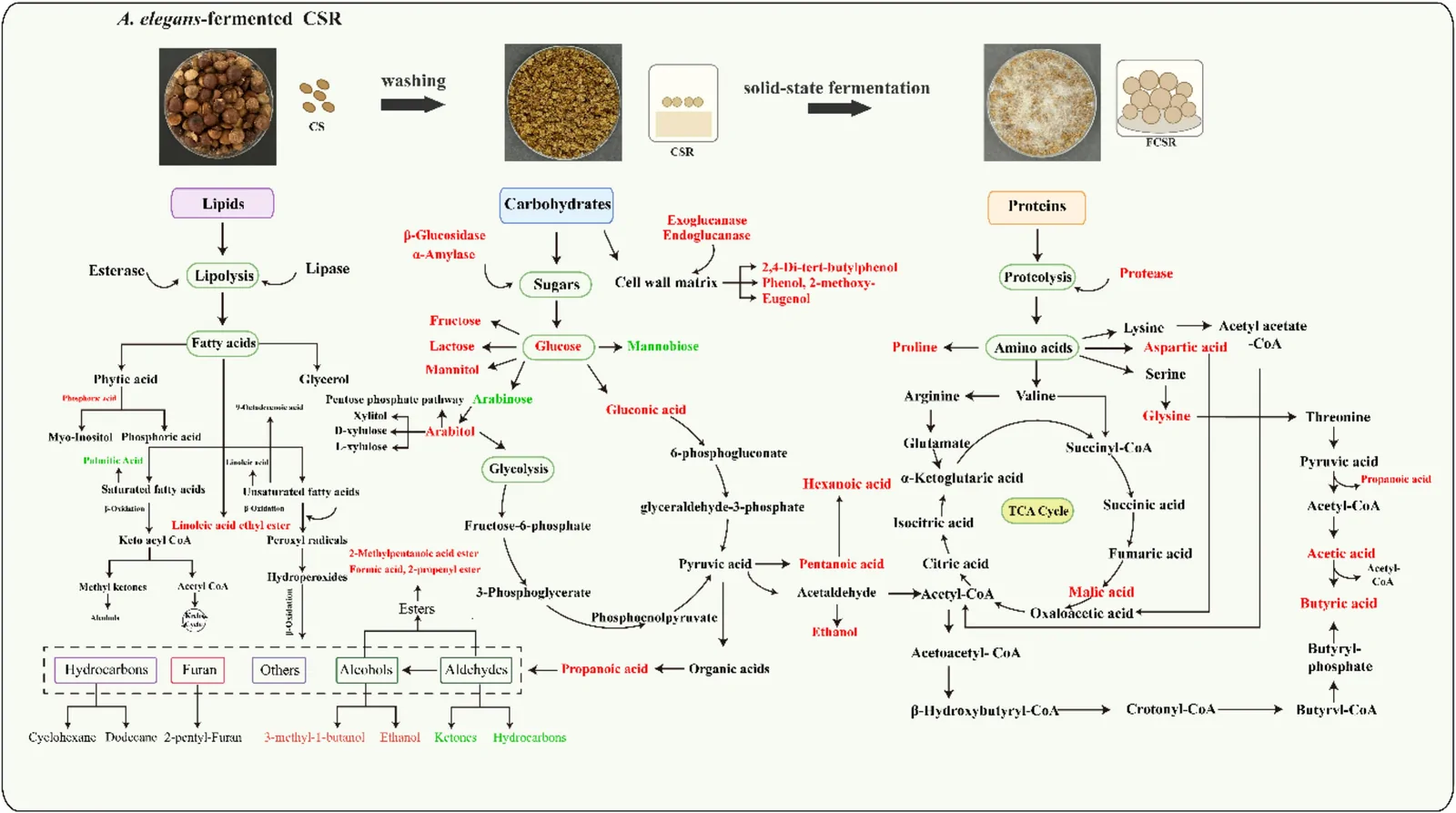
The potential metabolic pathways involved in fermentation. Upregulated metabolites: red color; Downregulated metabolites, green color; Undetected metabolites, black color. (For interpretation of the references to color in this figure legend, the reader is referred to the web version of this article.)

### 3.6 Effects of fermentation on the physicochemical properties of CSR

Fermentation markedly influenced the physicochemical properties of CSR including WSI, WHC, OHC, and CEC. WSI increased continuously with fermentation time, which may be largely explained by the liberation of reducing sugars, SDF, soluble proteins, and low-molecular-weight organic acids (Fig. 2E). Meanwhile, WHC, OHC, and CEC each exhibited an initial rise followed by a subsequent decline (Fig. 2F-G and Fig. S4). At the early stage, cellulase and other hydrolases are presumed to broke down the dense polysaccharide matrix of CSR, leading to a loosened structure and enhanced exposure of hydrophilic functional groups such as hydroxyl and carboxyl, which may in turn improve water/oil retention and cation binding (Li et al., 2022). This was supported by the results of FT-IR and XRD (Fig. 2H-I). The strengthened peak at 3400 cm^−1^ in FCSR, corresponding to -OH stretching, reflected greater hydroxyl availability, whereas the reduced relative crystallinity (RC) value pointed to structural disintegration of the fiber network. However, prolonged fermentation led to over-degradation of the porous fiber network structure, thereby weakening the WHC and OHC during late fermentation (Days 7–10). This was in line with the report of Sun et al. (2026). The high correlation among enzyme activities, nutrient content and physicochemical properties suggest that fungal enzymatic hydrolysis may have driven changes in nutrient composition, which in turn were associated with the observed changes in physicochemical properties (Fig. 2J). The above results indicated that moderate fermentation could improve the processing suitability of CSR as a food ingredient. These fermentation-induced changes in solubility and water/oil-holding capacity provide a basis for the noodle quality results described below.

### 3.7 Effects of CSR and FCSR on the sensory quality of noodles

Based on the fermentation-induced functional changes described above, FCSR was incorporated into wheat noodles at 1–6%, and its effects on sensory quality, cooking performance, structure, and starch digestibility were evaluated. As shown in Table 3, noodle color scores dropped considerably upon the addition of 4% CSR and FCSR, reflecting a trend toward darker product appearance (Fig. 4A). This was in line with the results of colorimeter analysis (Table S2). The reduced L* and b* values indicated diminished lightness and blue shift, whereas the increased a* value reflected a shift from white toward redder tones. This color change was attributed to Maillard reactions, in which reducing sugars and amino acids undergo chemical condensation during fermentation and subsequent heat treatment, generating brown-colored products that deepen the overall appearance (Wang et al., 2018). Both CSR and FCSR addition enhanced noodle elasticity, with FCSR exhibiting a more pronounced effect, owing to the high hydrophilicity of SDF, which hydrates to form a viscous colloidal network that facilitates starch–gluten cross-linking (Nakauma et al., 2008). While CSR reduced the aroma score, FCSR showed a higher aroma score, which is consistent with the fermentation-induced changes in volatile composition. However, the specific compounds responsible for this sensory difference were not identified. The taste score of noodles with 1–4% FCSR remained comparable to that of the control, suggesting that the acidity introduced by FCSR was mild and acceptable at the optimal addition level, and only became a limiting factor at 6%. This was further supported by the results of the pH variation in the steamed noodles (Fig. S5), which was due to the produced SCFAs and the other organic acids during fermentation. Sensory parameters including firmness and overall acceptability also decreased notably when CSR exceeded 2% or FCSR exceeded 4%, indicating that excessive supplement levels impaired noodle quality. Accordingly, the acceptable addition level was 2% for CSR and 4% for FCSR, indicating that fermentation improved the sensory tolerance of the residue in noodles.

**Table 3:** Sensory evaluation of noodles added with CSR and FCSR.

Sample	Content (%)	Color	Firmness	Elasticity	Adhesiveness	Aroma	Taste	Overall acceptability
CK	0	7.27 ± 0.51^Aa^	6.67 ± 0.65 ^Aa^	6.23 ± 0.67^Cd^	7.47 ± 0.76^Aa^	6.83 ± 0.52^Ba^	7.43 ± 0.99^Aa^	7.07 ± 0.51^Aa^
CSR	1	7.20 ± 0.65^a^	6.60 ± 0.49^ab^	6.33 ± 0.47^cd^	7.43 ± 1.41^a^	6.90 ± 0.30^a^	7.10 ± 0.75^a^	6.80 ± 0.87^ab^
	2	7.00 ± 0.63^a^	6.63 ± 0.60^ab^	6.70 ± 0.59^b^	7.63 ± 0.60^a^	6.67 ± 0.54^ab^	7.23 ± 0.67^a^	6.83 ± 0.82^ab^
	4	5.77 ± 0.56^b^	6.30 ± 0.78^b^	7.10 ± 0.30^a^	7.60 ± 0.92^a^	6.70 ± 0.64^ab^	6.73 ± 0.51^b^	6.47 ± 0.72^bc^
	6	4.87 ± 0.43^c^	5.90 ± 0.54^c^	6.57 ± 0.72^bc^	7.53 ± 0.76^a^	6.43 ± 0.50^b^	6.63 ± 0.48^b^	6.33 ± 0.94^c^
FCSR	1	7.20 ± 0.48 ^A^	6.73 ± 0.68 ^A^	6.87 ± 0.73^B^	7.70 ± 0.74 ^A^	7.17 ± 0.64^A^	7.50 ± 0.62 ^A^	7.20 ± 0.70 ^A^
	2	6.97 ± 0.71 ^A^	6.57 ± 0.56^A^	7.13 ± 0.62^AB^	7.80 ± 0.75^A^	7.23 ± 0.62 ^A^	7.40 ± 0.80 ^A^	6.93 ± 0.68 ^A^
	4	5.87 ± 0.43^B^	6.43 ± 0.72^A^	7.50 ± 0.85^A^	7.67 ± 0.83 ^A^	7.47 ± 0.56^A^	7.17 ± 0.45^AB^	6.87 ± 0.56^A^
	6	4.83 ± 0.58^C^	6.03 ± 0.71^B^	7.27 ± 0.73^A^	7.57 ± 0.67^A^	7.40 ± 0.61^A^	6.83 ± 0.64^B^	6.37 ± 0.71^B^

Means with different letters are significantly different (*p* < 0.05). The letters indicate the ordering of highness of parameter between different contents of the same CSR group, and “A and a” means the highest level.

**Fig. 4:**
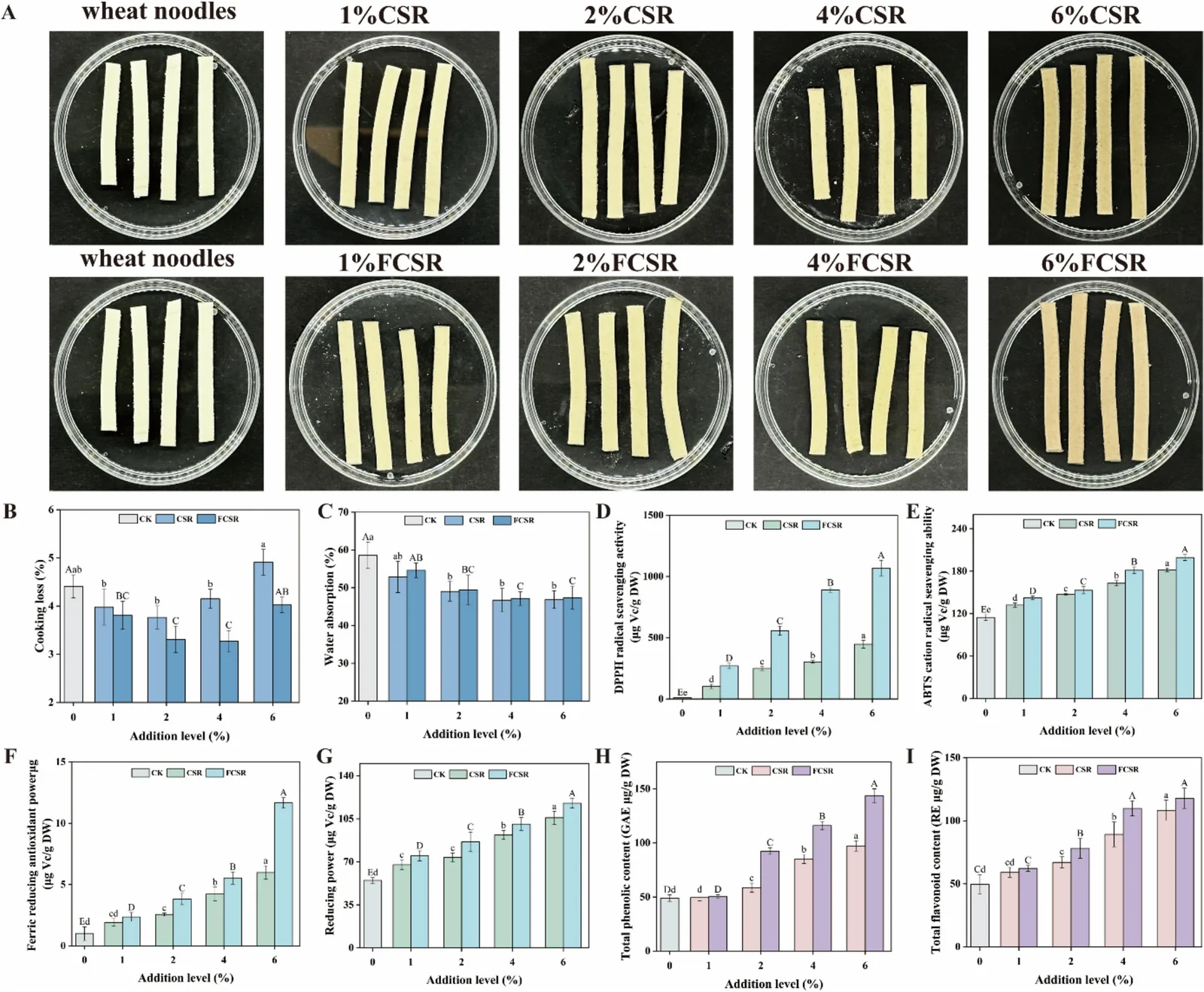
Effects of CSR and FCSR addition on the food quality, antioxidant activity and phenolic content of wheat noodles. (A) Appearance observation; (B) cooking loss; (C) water absorption; (D) DPPH radical scavenging activity; (E) ABTS•^+^ radical scavenging activity; (F) FRAP; (G) RP; (H) phenolic content; (I) flavonoid content.

### 3.8 Effects of CSR and FCSR on the cooking characteristics of noodles

Both adding CSR and FCSR at level below 4% reduced noodle cooking loss, with the lowest value (3.27%) recorded at 4% FCSR, supporting 4% FCSR as the optimal addition level (Fig. 4B). This reduction might be attributed to the presence of IDF, SDF, and phenolics in the supplements, which can cross-link with starch or gluten protein to generate compact network, thereby strengthening the entrapment of starch granule and restricting their leaching during cooking (Kong et al., 2026). Conversely, excessive supplementation tended to disrupt the noodle matrix, leading to increased cooking loss. Notably, FCSR-supplemented noodles exhibited lower cooking loss than CSR, possibly because fermentation exposed more functional groups within the IDF and SDF structure, facilitating stronger binding to starch granules (Meng et al., 2025). Meanwhile, the addition of CSR and FCSR led to a substantial decline in the water uptake (Fig. 4C). This was likely because SDF and phenolics contended with starch for the accessible water, restraining granule swelling and hydration, which was supported by the earlier work of Li et al. (2023), who reported reduced moisture absorption of the noodle product supplemented with mushroom-derived protein–polysaccharide complexes.

### 3.9 Effects of CSR and FCSR on the antioxidant capacities of noodles

As shown in Fig. 4D, both CSR and FCSR supplementation significantly enhanced DPPH radical-scavenging activity. For instance, the inclusion of 6% (w/w) CSR led to a 40.7-fold increase compared with control noodles. Consistent results were obtained for ABTS• + scavenging capacity, FRAP and RP (Fig. 4E-G). These improvements were attributable to the phenolic and flavonoid compounds present in the supplements (Fig. 4H-I, Kong et al., 2026). Notably, FCSR-enriched noodles consistently outperformed their CSR counterparts in all antioxidant assays, a finding consistent with Wang, Li, et al. (2025), who reported a direct correlation between higher phenolic and flavonoid contents and stronger radical-scavenging capacity. Furthermore, fermentation-induced structural disruption of SDF and IDF surfaces likely increased the specific surface area and exposed a greater number of reactive moieties and functional groups, thereby facilitating free-radical quenching activity (Jin et al., 2025). Given that increased antioxidative capacity has been associated with potential health benefits, FCSR supplement may represent a promising strategy for improving the functional quality of noodles.

### 3.10 Effects of CSR and FCSR on the *in vitro* starch digestibility of wheat noodles

Both CSR and FCSR addition significantly suppressed noodle starch hydrolysis in a dose-dependent manner (Fig. 5A-B). Native noodles were dominated by RDS, accounted for 40.48% of the total starch content (Fig. 5C), which is known to provoke sharp postprandial glucose spikes and heighten insulin resistance risk (Li et al., 2023). However, both CSR and FCSR addition effectively converted digestible starch into non-digestible fraction, as reflected by reduced RDS and increased RS content. According to Xiong et al. (2024) and Maibam et al. (2023), phenolics and dietary fibers especially SDF have been reported to inhibit amylase activity *in vitro* during digestion and bind with amylose during heating, potentially forming a physical shield that may hamper amylase attack and yields a smoother, denser noodle surface. RS, being fiber-like, has been reported to offer satiety and glycemic control and may therefore be beneficial for diabetic or obese populations (Zhang, Yu, et al., 2026; Zhang, Zhou, et al., 2026). FCSR outperformed CSR in transforming digestible starch into non-digestible forms, leading to a markedly lower hydrolysis rate. The digestion kinetic parameters, including C_∞_, k, and HI, exhibited an inverse relationship with RS content (Fig. 5D-F). The reduction in C_∞_ indicated that both CSR and FCSR supplementation substantially reduced *in vitro* starch digestibility, further supported by the diminished k value, which denotes the initial hydrolysis velocity. Given that HI serves as a proxy for pGI in this *in vitro* system, its decline corresponded to a markedly lower pGI for the fortified noodles (Fig. 5G). Notably, FCSR-enriched noodles yielded a greater reduction in pGI than their CSR counterparts, consistent with their comparatively slower *in vitro* hydrolysis kinetics, suggesting a potentially greater benefit for moderating postprandial glucose responses, which requires *in vivo* confirmation. Taking together the sensory acceptability in Section 3.7, cooking quality in Section 3.8 and starch digestibility results, 4% FCSR represented the optimal balance: lower levels (1–2%) did not significantly improve RS/pGI, whereas higher levels (6%) compromised mouthfeel without further benefit. Therefore, 4% FCSR was selected as the optimal threshold for noodle fortification.

**Fig. 5:**
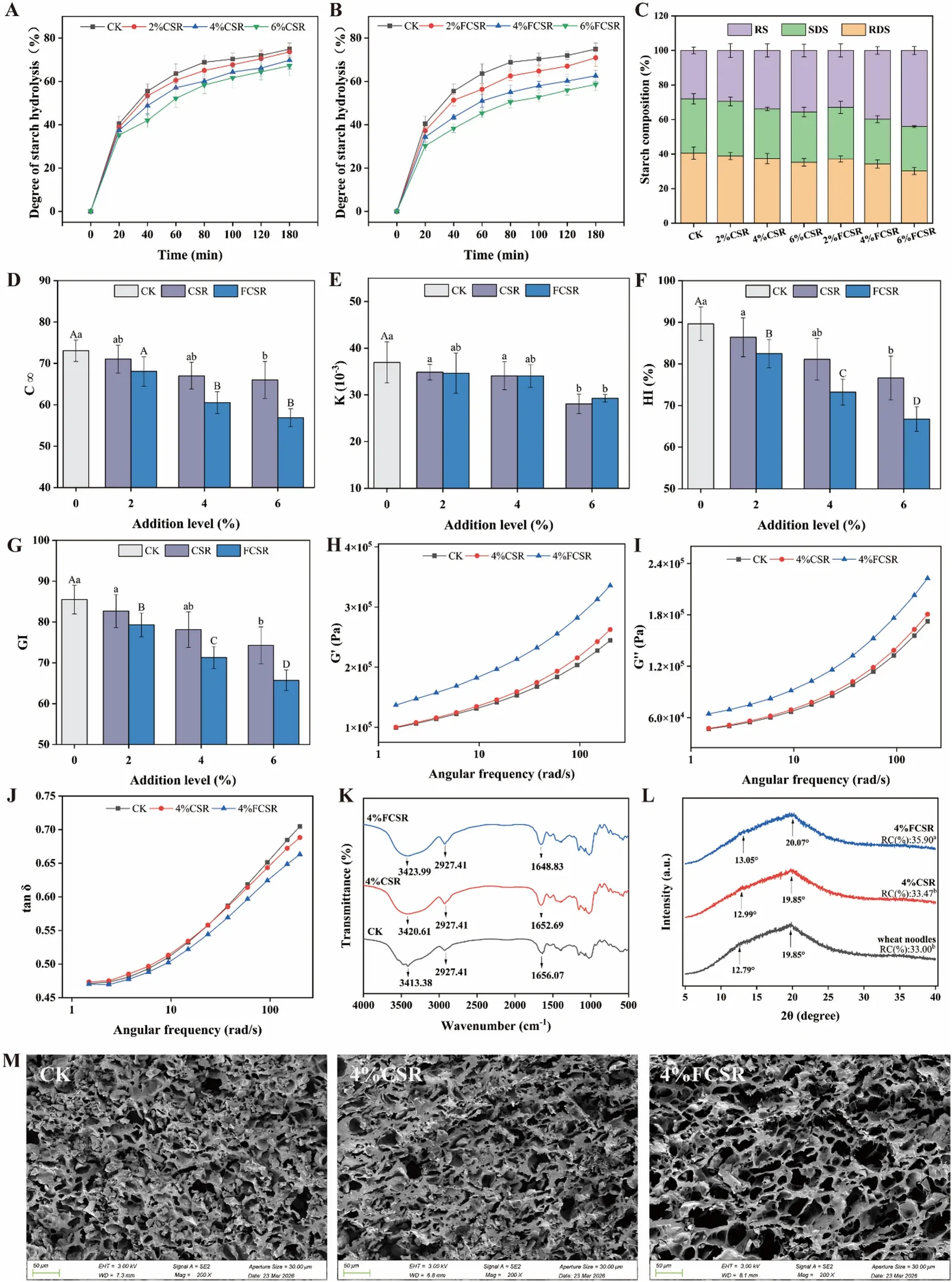
Effects of CSR and FCSR on the digestion and structure of noodles. (A) CSR starch hydrolysis; (B) FCSR starch hydrolysis; (C) starch compositions; (D) C∞; (E) K value; (F) HI value; (G) pGI value; (H) G′; (I) G″; (J) tan δ; (K) FT-IR analysis; (L) XRD analysis; (M) SEM observation.

### 3.11 Effects of CSR and FCSR on the structure of wheat noodles

Dough rheology depends critically on the structural soundness of the hydrated gluten network, which dictates processing and final quality (Tian et al., 2025). Fig. 5H-I showed that CSR and FCSR raised G′ and G″ *versus* the control, yet G′ remained consistently higher than G″, confirming no fundamental change in viscoelastic nature. Since G′ represents elastic storage and G″ viscous dissipation, the modulus enhancement implied that both CSR and FCSR reinforced dough elasticity either by promoting water loss within the gluten matrix or by acting as passive fillers dispersed throughout the viscoelastic network (Lin et al., 2021). The concomitant tan δ reduction reflects a more elastic, solid-like dough, probably due to intensified water competition with gluten (Fig. 5J, Liu, Song, et al., 2022). This pattern echoed the earlier findings of Sui et al. (2018) in bran-amended doughs. Interestingly, FCSR dough exhibited higher storage (G′) and loss (G″) moduli and lower tan δ than CSR and control dough. This might be associated with fermentation-related increases in SDF and phenolics, together with changes in the fiber structure. Reports have shown that phenolics and SDF especially fermented SDF can promote the three-dimensional network structure of dough *via* cross-linking with gluten proteins or starch molecules, consequently reinforcing both the elasticity and structural integrity of the gluten matrix (Jin et al., 2025; Kong et al., 2026; Zhang, Zhou, et al., 2026). FTIR spectra of CSR- and FCSR-supplemented noodles showed no detectable differences from the control, excluding any novel chemical incorporation (Fig. 5K). This concurs with Luo et al. (2022), who found fibril aggregates did not alter wheat starch's covalent structure. Therefore, CSR/FCSR–starch associations were attributable exclusively to intermolecular forces. The band near 3400 cm^−1^, attributed to O—H stretching and hydrogen bonding in pyranose rings, marks the thermodynamic origin of double-helix assembly during starch chain reassociation (Maibam et al., 2023). Its enhanced intensity after CSR/FCSR addition indicated stronger hydrogen bonding. The CSR and FCSR addition also increased the RC values of noodles (Fig. 5L). As documented by Wang et al. (2017), the crystalline supramolecular architecture of starch confers long-range ordered structures that effectively impede enzymatic digestion by restricting both enzyme access and substrate binding. Supporting evidence from SEM indicated that FCSR addition prominently facilitated the formation of a better-organized and more porous microarchitecture in the noodle matrix (Fig. 5M). Taken together, the FCSR-induced reduction in pGI value may be attributed to the possible α-amylase inhibition and the physical barrier formed by cross-linking interactions among FCSR, starch, and gluten proteins.

## 4 Conclusion

This study demonstrated that *A. elegans* fermentation effectively upgraded CSR into a functional food ingredient by enhancing its nutritional profile, phenolic content, antioxidant activity, while modifying its volatile composition and physicochemical properties. Notably, fermentation increased the SDF content by 2.62-fold and phenolic content by 79.44% compared with native CSR. When used as a functional ingredient, 4% FCSR provided the best balance between noodle sensory acceptability and glycemic benefit, increasing RS by 42.18% and reducing pGI by 16.57%, while improving dough rheology and microstructure. These benefits are most likely attributable to enzymatic liberation of bioactive compounds and structural modification of dietary fibers, although the underlying mechanisms were not directly verified. This study lacks *in vivo* validation of glycemic and health effects, and the applicability of FCSR to other food systems beyond noodles remains untested. Future work should focus on animal or human trials to confirm health benefits, evaluate consumer sensory acceptability in large-scale consumer trials, and investigate the industrial-scale fermentation process to facilitate commercial application of FCSR in functional foods.

## CRediT authorship contribution statement

**Zhenduo Gao:** Writing – original draft, Investigation. **Guanyu Fang:** Software, Methodology, Data curation. **Yaoyu Xu:** Methodology, Formal analysis. **Yixue Zhou:** Methodology, Investigation. **Fenghua Wu:** Supervision, Software. **Zhiping He:** Supervision, Data curation. **Deyang Lin:** Software, Investigation. **Baiyi Lu:** Writing – review & editing, Conceptualization. **Xingquan Liu:** Writing – review & editing, Data curation, Conceptualization. **Yongzhu Zhang:** Writing – review & editing, Funding acquisition, Data curation.

## Ethics declaration

### Informed consent and patient details

Written informed consent to take part in the study and to publish the article has been obtained from all participants or their legal representatives. The privacy rights of participants have been observed.

### Studies in human

This study was performed in compliance with relevant laws, regulatory frameworks and guidelines where the research took place. This study was approved by the Zhejiang Agriculture and Forestry University Committee of Life Sciences and Medical Ethics (Approval No. No. SMYX-2026-013).

## Declaration of competing interest

The authors declare that they have no known competing financial interests or personal relationships that could have appeared to influence the work reported in this paper.

## Data Availability

Data will be made available on request.

## References

[bb0005] Akhtar M., Chen Y., Ma Z., Zhang X., Shi D., Khan J.A., Liu H. (2022). Gut microbiota-derived short chain fatty acids are potential mediators in gut inflammation. Animal Nutrition.

[bb0010] Canoy T.S., Wiedenbein E.S., Bredie W.L.P., Meyer A.S., Wösten H.A.B., Nielsen D.S. (2024). Solid-state fermented plant foods as new protein sources. Annual Review of Food Science and Technology.

[bb0015] Chen Y., Wang Y., Chen J., Tang H., Wang C., Li Z., Xiao Y. (2020). Bioprocessing of soybeans (*Glycine max* L.) by solid-state fermentation with *Eurotium cristatum* YL-1 improves total phenolic content, isoflavone aglycones, and antioxidant activity. *RSC* Advances.

[bb0020] Choi C.W., Choi Y.H., Cha M.R., Yoo D.S., Kim Y.S., Yon G.H., Ryu S.Y. (2010). Yeast α-glucosidase inhibition by isoflavones from plants of *Leguminosae* as an in vitro alternative to acarbose. Journal of Agricultural and Food Chemistry.

[bb0025] Cui Y., Ding C., Liu Q., Xi J., Meng J., Dong T., Jia T., Du T. (2024). Effects of solid-state fermented wheat bran on inflammation level, serum lipid profiles and intestinal microbiota in Kunming mice. *Food* Bioscience.

[bb0030] Di Cagno R., Surico R.F., Paradiso A., De Angelis M., Salmon J.C., Buchin S., Gobbetti M. (2009). Effect of autochthonous lactic acid bacteria starters on health-promoting and sensory properties of tomato juices. International Journal of Food Microbiology.

[bb0035] Ding Y., Wan J., Liu C., Shi X., Xia X., Prakash S., Zhang X. (2020). Retrogradation properties and *in vitro* digestibility of wild starch from *Castanopsis sclerophylla*. Food Hydrocolloids.

[bb0040] Fawole O.A., Opara U.L. (2014). Physicomechanical, phytochemical, volatile compounds and free radical scavenging properties of eight pomegranate cultivars and classification by principal component and cluster analyses. British Food Journal.

[bb0045] Gill S.K., Rossi M., Bajka B., Whelan K. (2021). Dietary fibre in gastrointestinal health and disease. Nature Reviews Gastroenterology and Hepatology.

[bb0050] Granfeldt Y., Björck I., Drews A., Tovar J. (1992). An *in vitro* procedure based on chewing to predict metabolic response to starch in cereal and legume products. European Journal of Clinical Nutrition.

[bb0055] Huang R., Huang K., Guan X., Zhang J., Zhang P. (2022). Incorporation of defatted quinoa flour affects *in vitro* starch digestion, cooking and rheological properties of wheat noodles. Journal of Cereal Science.

[bb0060] Jin Y., Zhao Y., Azi F., Xue W., Cheng J., Qian J., Tang X., Xie D., Zheng J., Jin P., Yin L. (2025). Characterization of soluble dietary fiber from *R. Oligosporus*-fermented sweetpotato residues and its effects on the quality attributes of noodles. LWT--Food Science and Technology.

[bb0065] Kong F., Li Y., Lu H., Ding Y. (2026). Comparative effects of wheat bran modifications by microwaving, steam explosion and solid-state fermentation on reconstituted dough properties and corresponding noodles qualities. Food Chemistry*: X*.

[bb0070] Li H., Hao Y., Dai Y., Chen Z., Ping Y., Zhao B. (2023). Effects of protein-polysaccharide extracted from *Auricularia auricula-judae* mushroom on the quality characteristics of Chinese wheat noodles. LWT - Food Science and Technology.

[bb0075] Li N., Wang S., Wang T., Liu R., Zhi Z., Wu T., Sui W., Zhang M. (2022). Valorization of wheat bran by three fungi solid-state fermentation: Physicochemical properties, antioxidant activity and flavor characteristics. Foods.

[bb0080] Lin S., Jin X., Gao J., Qiu Z., Ying J., Wang Y., Dong Z., Zhou W. (2021). Impact of wheat bran micronization on dough properties and bread quality: Part I: Bran functionality and dough properties. Food Chemistry.

[bb0085] Liu D., Song S., Tao L., Yu L., Wang J. (2022). Effects of common buckwheat bran on wheat dough properties and noodle quality compared with common buckwheat hull. LWT - Food Science and Technology.

[bb0090] Liu Y., Gu P., Laaksonen O., Wei B., Zhu Y., Zhang B., Zhu B., Li H. (2022). Lactic acid bacteria incubation and aging drives flavor enhancement of goji berry juice. Journal of Food Composition and Analysis.

[bb0095] Luo J., Zhai Y., Wu J., Wang F., Liu Y., Li X. (2022). Mechanism governing the rice glutelin fibrils on inhibition of in vitro wheat starch digestion. Food Hydrocolloids.

[bb0100] Maibam B.D., Nickhil C., Deka S.C. (2023). Preparation, physicochemical characterization, and in vitro starch digestibility on complex of *Euryale ferox* kernel starch with ferulic acid and quercetin. International Journal of Biological Macromolecules.

[bb0105] Melgarejo P., Calín-Sánchez Á., Vázquez-Araújo L., Hernández F., Martínez J.J., Legua P., Carbonell-Barrachina Á.A. (2011). Volatile composition of pomegranates from 9 Spanish cultivars using headspace solid phase microextraction. Journal of Food Science.

[bb0110] Meng W., Hu M., Zhang P., Wang J., Yuan Z., Wang F., Li S. (2025). Efficient conversion of insoluble dietary fiber to soluble dietary fiber by *Bacillus subtilis* BSNK-5 fermentation of okara and improvement of their structural and functional properties. Food Chemistry.

[bb0115] Nakauma M., Funami T., Noda S., Ishihara S., Al-Assaf S., Nishinari K., Phillips G.O. (2008). Comparison of sugar beet pectin, soybean soluble polysaccharide, and gum arabic as food emulsifiers. 1. Effect of concentration, pH, and salts on the emulsifying properties. Food Hydrocolloids.

[bb0120] Ren X., Chen Q., Wang F., Guo H., Wang Y., Gu F. (2025). Dynamic changes in nutritions and antioxidant activities of germinated black bean based on solid-state fermentation of *Ganoderma oregonense*. Innovative Food Science & Emerging Technologies.

[bb0125] Sui W., Xie X., Liu R., Wu T., Zhang M. (2018). Effect of wheat bran modification by steam explosion on structural characteristics and rheological properties of wheat flour dough. Food Hydrocolloids.

[bb0130] Sun Y., Zheng Y., Liu N., Qier M., Qi J., An X. (2026). Mechanistic insights into grifola frondosa-driven fermentation of rice-wheat bran: Metabolomic profiling, molecular dynamics, and enhanced antioxidant efficacy. Food Chemistry: X.

[bb0135] Tian G., Li M., Xing S., He L., Li C., Qiao S., Yuan Y., Luo T. (2025). Modified okara improves noodle properties: Effects on digestibility, structure, and functionality. Current Research in Food Science.

[bb0140] Wang L., Zhang B., Xiao J., Huang Q., Li C., Fu X. (2018). Physicochemical, functional, and biological properties of water-soluble polysaccharides from *Rosa roxburghii* Tratt fruit. Food Chemistry.

[bb0145] Wang Q., Li L., Hu L., Cao Y., Cao Y., Gao J., Zhang H., Zhou F., Wu Y., Li S., Wang R. (2025). Investigation of the solid-state fermentation of *Astragalus membranaceus* residue with *Cordyceps militaris*: Effects on phenolic profile and in vitro neuroprotective activity. Food Chemistry.

[bb0150] Wang S., Wang S., Liu L., Wang S., Copeland L. (2017). Structural orders of wheat starch do not determine the in vitro enzymatic digestibility. Journal of Agricultural and Food Chemistry.

[bb0155] Wang Y., Liang H., Hu Z., Chen L., Zhu L., Zhuang K., Ding W., Shen Q. (2025). Evaluation of flavor properties in rice bran by solid-state fermentation with yeast. Food Chemistry*: X*.

[bb0160] Wei C., Ge Y., Liu D., Zhao S., Wei M., Jiliu J., Hu X., Quan Z., Wu Y., Su Y., Wang Y. (2022). Effects of high-temperature, high-pressure, and ultrasonic treatment on the physicochemical properties and structure of soluble dietary fibers of millet bran. *Frontiers in* Nutrition.

[bb0165] Wu H., Rui X., Li C., Xia X., Dong M. (2018). Improved protein nutritional quality of whole oat grains by solid state fermentation with mixed strains. Food Science.

[bb0170] Xia Q., Li Y. (2018). Ultra-high-pressure effects on color, volatile organic compounds and antioxidants of wholegrain brown rice (*Oryza sativa* L.) during storage: A comparative study with high-intensity ultrasound and germination pretreatments. Innovative Food Science and Emerging Technologies.

[bb0175] Xiao Y., He C., Chen Y., Ho C.T., Wu X., Huang Y., Liu Z. (2022). UPLC–QQQ–MS/MS-based widely targeted metabolomic analysis reveals the effect of solid-state fermentation with *Eurotium cristatum* on the dynamic changes in the metabolite profile of dark tea. Food Chemistry.

[bb0180] Xiong S., Tao P., Yu Y., Wu W., Li Y., Chen G., Si J., Yang H. (2024). Effect of *Polygonatum cyrtonema* Hua polysaccharides on gluten structure, in vitro digestion and shelf-life of fresh wet noodle. International Journal of Biological Macromolecules.

[bb0185] Yin L., Liu Z., Lu X., Cheng J., Lu G., Sun J., Yang H., Guan Y., Pang L. (2023). Analysis of the nutritional properties and flavor profile of sweetpotato residue fermented with *Rhizopus oligosporus*. LWT--Food Science and Technology.

[bb0190] Yin L., Zhang Y., Wu H., Wang Z., Dai Y., Zhou J., Liu X., Dong M., Xia X. (2020). Improvement of the phenolic content, antioxidant activity, and nutritional quality of tofu fermented with *Actinomucor elegans*. LWT--Food Science and Technology.

[bb0195] Zhang Y., Liu M., Wu F., Li J., Azi F., Yu K., He Z., Wang P., Wu C., Lu B., Liu X. (2024). Fungal fermentation improves the nutritional quality, flavor characteristic and physicochemical property of highland barley bran. Journal of Cereal Science.

[bb0200] Zhang Y., Wei R., Azi F., Jiao L., Wang H., He T., Liu X., Wang R., Lu B. (2022). Solid-state fermentation with *Rhizopus oligosporus* RT-3 enhanced the nutritional properties of soybeans. *Frontiers in* Nutrition.

[bb0205] Zhang Y., Yu K., Fang G., Lu B., Azi F., Gao Z., Zhou Y., Wu F., Hu H., Sun X., Liu X. (2026). Improvement of the nutritional composition, flavor characteristic, and functional activity of brown rice bio-processed with *R. Oligosporus*. Journal of Cereal Science.

[bb0210] Zhang Y., Zhou Y., Fang G., Azi F., Gao Z., Xu Y. (2026). Interaction between epicatechin and wheat starch and its effect on the food quality of noodles. Journal of Food Process Engineering.

[bb0215] Zhao Y., Liu D., Zhang J., Shen J., Cao J., Gu H., Cui M., He L., Chen G., Liu S., Shi K. (2024). Improving soluble phenolic profile and antioxidant activity of grape pomace seeds through fungal solid-state fermentation. Foods.

