# Supplementary material for: Actinomucor elegans-fermented Castanopsis sclerophylla residue as a functional ingredient for low-glycemic wheat noodles: fermentation-induced functionalization and noodle quality
Source: Food Chem X. 2026 Oct 28;40:104724. doi: 10.1016/j.fochx.2026.104724 (PMC13742510; doi:10.1016/j.fochx.2026.104724)
Supplement: Supplementary file 1 [file mmc1.docx]

**Fig. S1** Pearson correlation analysis among fungal enzyme, phenolics and antioxidant activity. ABTS⁺, 2,2’-azino-bis(3-ethylbenzothiazoline-6-sulfonic acid); DPPH, 2,2-diphenyl-1-picrylhydrazyl; FRAP, ferric reducing antioxidant power; RP, reducing power; TPC, total phenolic content; TFC, total flavonoid content. Asterisks indicate significance: * *p*≤0.05; ** *p*≤0.01; *** *p*≤0.001.

**Fig. S2** The PCA analysis of metabolite composition. (A) 2D score plot, (B) 3D score plot, and (C) PCA with PERMANOVA results (n = 3 per group). CSR, *Castanopsis sclerophylla* residue; FCSR, fermented CSR

**Fig. S3** The cluster dendrogram of metabolite composition. CSR, *Castanopsis sclerophylla* residue; FCSR, fermented CSR

**Fig. S4** The changes of cation exchange capacity during fermentation. Different lowercase letters above the bars indicate significant differences among fermentation times (*p*<0.05).

**Fig. S5** The pH value of the steamed noodles. *p*<0.05 was considered as the signiﬁcant level. The lowercase superscript letters were used to show the signiﬁcant difference between CSR and CK where “a” means the highest yield. The uppercase superscript letters were used to show the signiﬁcant difference between FCSR and CK. CSR, *Castanopsis sclerophylla* residue; FCSR, fermented CSR; CK, control noodles.

**Table S1** Sensory evaluation of CSR and FCSR

| Fermentation (d) | Color | Flavor | Taste | Overall acceptability |
| --- | --- | --- | --- | --- |
| 0 | 1.77±0.12^b^ | 1.93±0.21^c^ | 1.07±0.06^d^ | 1.37±0.21^c^ |
| 1 | 1.80±0.10^b^ | 2.20±0.26^c^ | 1.37±0.06^c^ | 1.63±0.23^bc^ |
| 3 | 2.17±0.32^a^ | 3.90±0.10^b^ | 1.90±0.17^b^ | 1.93±0.25^b^ |
| 5 | 2.23±0.12^a^ | 4.40±0.20^a^ | 2.40±0.10^a^ | 2.47±0.21^a^ |
| 7 | 1.70±0.17^b^ | 3.53±0.25^b^ | 2.43±0.21^a^ | 1.83±0.31^b^ |

Different lowercase superscript letters within the same column indicate significant differences (*p*<0.05).

**Table S2** The colorimeter analysis of noodles added with CSR and FCSR

| Sample | Content  (%) | L* | a* | b* | Whiteness |
| --- | --- | --- | --- | --- | --- |
| CK | 0 | 76.74±0.07^Aa^ | 0.27±0.01 ^Aa^ | 15.37±0.03^Aa^ | 72.12±0.07^Aa^ |
| CSR | 1 | 73.12±0.11^b^ | 2.82±0.01^b^ | 14.37±0.03^b^ | 69.39±0.10^b^ |
|  | 2 | 70.24±0.10^c^ | 3.68±0.10^c^ | 14.22±0.08^b^ | 66.81±0.07^c^ |
|  | 4 | 65.67±0.13^d^ | 4.05±0.02^d^ | 14.14±0.06^b^ | 62.65±0.11^d^ |
|  | 6 | 60.13±0.20^e^ | 5.05±0.04^e^ | 14.18±0.02^b^ | 57.39±0.18^e^ |
| FCSR | 1 | 73.10±0.02^B^ | 2.52±0.03^B^ | 14.37±0.11^B^ | 69.40±0.07^B^ |
|  | 2 | 70.13±0.07^C^ | 3.76±0.10^C^ | 14.39±0.05^B^ | 66.63±0.06^C^ |
|  | 4 | 65.13±0.14^D^ | 4.12±0.06^D^ | 14.39±0.04^B^ | 62.05±0.12^D^ |
|  | 6 | 59.06±0.41^E^ | 5.41±0.06^E^ | 14.25±0.06^B^ | 56.31±0.37^E^ |

*p*<0.05 was considered as the signiﬁcant level. The lowercase superscript letters were used to show the signiﬁcant difference between CSR and CK for the same indicators where “a” means the highest yield. The uppercase superscript letters were used to show the signiﬁcant difference between FCSR and CK for the same indicators. CSR, *Castanopsis sclerophylla* residue; FCSR, fermented CSR; CK, control noodles.
